# Hypoxia induces chemoresistance of esophageal cancer cells to cisplatin through regulating the lncRNA-EMS/miR-758-3p/WTAP axis

**DOI:** 10.18632/aging.203062

**Published:** 2021-06-03

**Authors:** Zi-Jiang Zhu, Yao Pang, Gang Jin, Hong-Yi Zhang, Wen-Hao Wang, Jia-Wei Liu, Guang-Xin Tuo, Peng Wu, Yi Yang, Ze-Quan Wang, Kui Wang

**Affiliations:** 1 Department of Thoracic Surgery 2, Gansu Provincial People's Hospital, Lanzhou 730000, China; 2 School of Clinical Medicine, Gansu University of Traditional Chinese Medicine, Lanzhou 730000, China; 3 School of Clinical Medicine, Ningxia Medical University, Ningxia 750004, China

**Keywords:** hypoxia, chemoresistance, esophageal cancer, EMS, miR-758-3p

## Abstract

Hypoxia contributes significantly to the development of chemoresistance of many malignancies including esophageal cancer (EC). Accumulating studies have indicated that long non-coding RNAs play important roles in chemotherapy resistance. Here, we identified a novel lncRNA-EMS/miR-758-3p/WTAP axis that was involved in hypoxia-mediated chemoresistance to cisplatin in human EC. Hypoxia induced the expressions of lncRNA EMS and WTAP, and reduced the expression of miR-758-3p in EC cell line ECA-109. In addition, the expressions of EMS and WTAP were required for the hypoxia-induced drug resistance to cisplatin in EC cells, while overexpression of miR-758-3p reversed such chemoresistance. The targeting relationships between EMS and miR-758-3p, as well as miR-758-3p and WTAP, were verified by luciferase-based reporter assays and multiple quantitative assays after gene overexpression/knockdown. Moreover, we found significant correlations between tumor expressions of these molecules. Notably, higher levels of EMS/WTAP, or lower levels of miR-758-3p in tumors predicted worse survivals of EC patients. Furthermore, in a xenograft mouse model, targeted knockdown of EMS and WTAP in ECA-109 cells markedly attenuated the resistance of tumors to cisplatin treatments. Our study uncovers a critical lncRNA-EMS/miR-758-3p/WTAP axis in regulating hypoxia-mediated drug resistance to cisplatin in EC.

## INTRODUCTION

As a common type of cancer, esophageal cancer (EC) ranks as the 6th most lethal malignant tumor worldwide with approximately 572, 034 new cases in 2018 [1]. Because of the lack of efficient early detection approaches for tumor-specific symptoms, as well as the tumor’s extremely aggressive nature, EC is often diagnosed at a late stage, resulting in only around 20% of patients can survive for 5 years after diagnosis [2]. Therefore, better understanding the molecular mechanisms and screening biomarkers of EC are critical in EC treatments. Currently, the chemotherapy has been considered as an effective and well-established treatment approach for EC. For example, cisplatin (Cis-diammine-dichloro-platinum II, DDP), an organometallic platinum compound, was discovered to display potent anti-neoplastic effects on tumor cells in 1980s, and is now still being used as first-line treatment option for patients diagnosed with EC [3]. However, EC patients are still able to develop recurrent malignancies that tend to display a more aggressive phenotype because of the inherent ability of EC cells to become chemo-resistant. The chemotherapy resistance of EC to DDP is usually associated with more aggressive cancer phenotypes, and till now, reliable biomarkers that can be used to evaluate the responses of EC patients to such therapies are still in lack [4].

The development of drug resistance in cancer involves the alterations of the tumor microenvironment, and the underlying mechanisms have not been fully understood [5]. Accumulating studies from both bench and bed-side researches have demonstrated the role of hypoxia in tumor initiation and development, and pinpointed that hypoxia contributes significantly to the development of resistance to chemotherapy in cancers including EC [6]. Exposure of cancer cells to hypoxia leads to the expressions of many hypoxia-inducible genes that contribute significantly to the resistance to anticancer agents, such as hypoxia-inducible transcription factor (HIF), a dimeric protein consisting of a constitutively active subunit (HIF-1β) and an oxygen-sensitive subunit (HIF-1α) [7]. The targets of HIF gene are involved in many biological processes in promoting the growth and malignant phenotypes of cancer cells including cell survival, proliferation, extracellular matrix (ECM) remodeling, and epithelial-to-mesenchymal transition [8]. In addition, accumulating hypoxia-inducible genes, such as the multidrug resistance 1 protein (MDR1/ABCB1) and the ATP binding cassette (ABC) transporter family, have been identified to contribute to the resistance to anticancer agents [9, 10]. Furthermore, other mechanisms including a lack of drug induced senescence in hypoxic tumor cells, induction of autophagy by hypoxia, and driving an immunosuppressive phenotype through HIF-1α, have been proposed to contribute to hypoxia mediated chemotherapy resistance in cancers [11–13].

Previous investigations have identified an association between chemoresistance and the aberrant expression of many hypoxia mediated genes, which places HIF-1α as a widely accepted therapeutic target for various cancers. However, direct alteration of a single gene often exerts limited impacts in overcoming chemoresistance due to that normally multiple regulated signaling pathways are involved in malignant progression [14]. Thus, further understanding the molecular mechanisms underlying the chemoresistance of EC can ultimately improve the effectiveness of DDP treatments in minimizing the risk of recurrence in patients. Recently, the long non-coding RNAs (lncRNAs) have been found to play an emerging role in cancer progression. LncRNAs are endogenous cellular RNAs with a length of more than 200 nucleotides, and they do not encode protein. They are transcribed by RNA polymerase II, and share the similar structure as mRNA with a 3’ polyA tail and a 5’ cap [15, 16]. LncRNAs are shown to participate in the biological processes that influence gene expressions [17]. Increasing studies have suggested that the aberrant lncRNA expression is implicated in the initiation and development of various cancers [18]. Moreover, many lncRNAs are considered as potential novel biomarkers for cancer diagnosis and prognosis, and some of them are good therapeutic targets [19]. Importantly, recent studies have indicated that some lncRNAs play critical roles in chemoradiotherapy resistance in EC. For example, Zhou et al*.* found that compared with the parent cell lines, cisplatin-resistant EC cells had dysregulated expression of three lncRNAs (AFAP1-AS1, UCA1, and HOTAIR), and they further identified the correlations between tumor expression levels of these lncRNAs and the survivals of EC patients [20]. In addition, another lncRNA TUSC7 was identified to promote EC apoptosis and inhibit chemotherapy resistance via the miR-224/DESC1 axis [21]. However, only very few lncRNAs have been well-studied for their functions and mechanisms in EC chemoresistance so far.

Recently, a novel lncRNA named E2F1 messenger RNA (mRNA) stabilizing factor (EMS) was revealed to be able to connect c-Myc to cell cycle regulation and oncogenesis through modulating the stability of E2F1 mRNA in lung cancers [22]. However, the oncogenic roles of EMS have not been elucidated in other types of human cancers till now. In this study, we investigated whether and how EMS was involved in resistance of EC cells to DDP under the normoxic and hypoxic conditions. Through multiple molecular and cellular assays, we identified a novel lncRNA-EMS/miR-758-3p/WTAP axis in controlling the proliferation and survival of human EC cells upon DDP chemotherapy in the context of both the *in vitro* EC cell culturing and *in vivo* xenograft tumor growth in mice. This study suggests that EMS is a promising biomarker for diagnosis, prognosis of EC, and also an attractive novel target for treatment of EC.

## RESULTS

### Hypoxia induces EMS expression in esophageal carcinomas, which promotes cancer cell drug resistance against DDP

**Figure 1:**
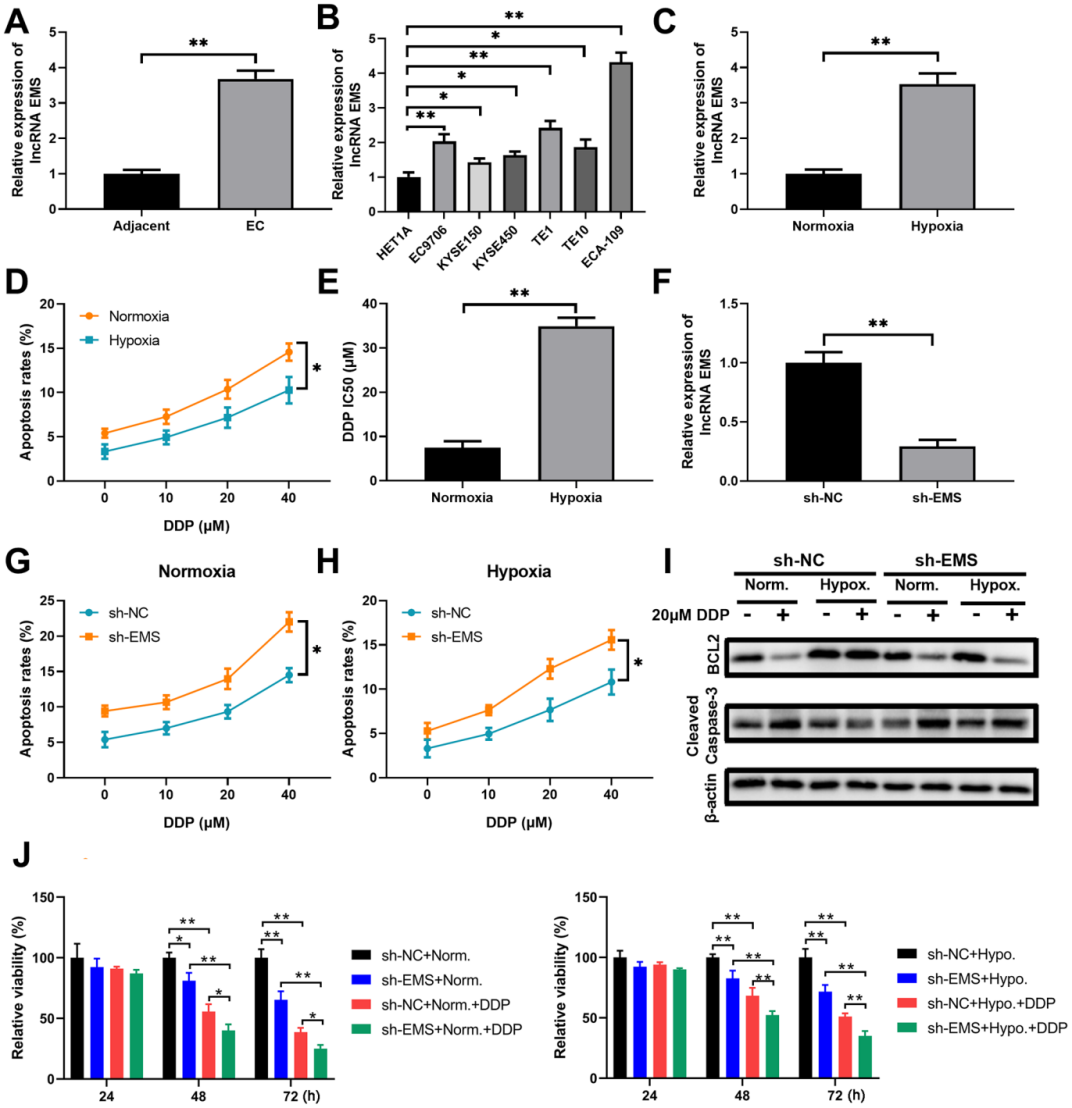
**LncRNA EMS is more expressed in esophageal carcinomas and contributes to cancer cell drug resistance against DDP.** (**A**) The expression levels of EMS in esophageal cancer tissues and the adjacent normal tissues were evaluated by RT-qPCR. n=20 for each group. (**B**) The expression levels of EMS in normal esophageal epithelial HET1A cell and multiple esophageal cancer cell lines (EC9706, ECA-109, KYSE150, KYSE450, TE1 and TE10) were determined by RT-qPCR. n=5 for each group. (**C**) The expression levels of EMS in ECA-109 cells under hypoxic condition and normoxic condition were determined by RT-qPCR. n=5 for each group. (**D**) The apoptosis rates (% of Annexin V-positive cells) of ECA-109 cells cultured under normoxic and hypoxic conditions in the presence of DDP at the indicated doses for 24 hours were determined by flow cytometry. (**E**) ECA-109 cells under hypoxic condition had significantly higher DDP IC50 values than the cells under normoxic condition. (**F**) qPCR results show the knockdown efficiency of EMS-shRNA in ECA-109 cells. n=5 for each group. (**G**, **H**) The apoptosis rates of control ECA-109 cells (sh-NC) and EMS silenced ECA-109 cells (sh-EMS) in response to the indicated concentrations of DDP under normoxic (**G**) and hypoxic (**H**) conditions were evaluated by flow cytometry. n=5 for each group. (**I**, **J**) Control (sh-NC) and EMS silenced (sh-EMS) ECA-109 cells were treated with 20 μM DDP or the vehicle under normoxic or hypoxic condition, and the BCL-2 and cleaved caspase-3 protein levels at 48 hours after treatments were determined by western blot (**I**). Cell proliferation under the indicated conditions for 4 days were measured by CCK-8 assays (**J**). n=5 for each group.

To identify the potential contribution of the lncRNA EMS in EC progression, we quantitated the relative transcript level of EMS in primary tumor tissues and human EC cell lines. The qPCR analyses demonstrated that EMS expression was markedly increased (around 3.5-fold) in the EC tumors than that in the adjacent normal tissues (Figure 1A). In compared with the normal esophageal epithelial HET1A cells, all the human EC cell lines, including ECA-109, EC9706, TE1, TE10, KYSE150 and KYSE450, had significantly higher levels of EMS transcript (Figure 1B). In particularly, ECA-109 cells displayed over 4-fold EMS expression, and this cell line with the highest abundance of EMS expression was selected as a model EC cell line for further studies.

It is well documented that many genes can be induced by hypoxia, which contributes to tumor progression and treatment resistance [23]. In order to figure out whether EMS is a hypoxia and drug-resistance associated lncRNA, we treated ECA-109 cells with the common chemotherapeutic drug cisplatin (DDP), and evaluated the impacts of EMS knockdown on cell apoptosis and proliferation under the hypoxic or normoxic conditions. We found that hypoxia alone significantly induced EMS expression in ECA-109 cells, as shown by more than 3-fold increase of EMS transcript in hypoxia-exposed cells when compared to the cells under normoxic condition (Figure 1C). In addition, a short time (24 hours) challenge with DDP at various concentrations (10/20/40 μM) dose-dependently increased apoptosis rates of ECA-109 cells. However, the cells under the hypoxic condition always had a lower apoptosis rate than the cells under the normoxic condition when cells were treated with the same dose of DDP (Figure 1D and Supplementary Figure 1A), and hypoxia rendered a significantly higher DDP IC_50_ than normoxia (Figure 1E). These results suggested that hypoxia did promote the drug-resistance against DDP in ECA-109 cells.

To further study the role of EMS in hypoxia-mediated DDP-resistance, we knocked down EMS in ECA-109 cells (Figure 1F and Supplementary Figure 1B) and compared the apoptosis rates and proliferation index between control cells and EMS-knockdown cells. Under either normoxic condition (Figure 1G and Supplementary Figure 1C) or hypoxic condition (Figure 1H and Supplementary Figure 1D), EMS silence markedly increased apoptosis rates of cells treated with DDP at various concentrations. This was also supported by reduced BCL-2 expressions and increased cleaved caspase-3 levels (Figure 1I), as well as suppressed cell proliferation (Figure 1J). It is worth noting that under the hypoxic condition, 20μM DDP treatment did not significantly alter the protein levels of BCL-2 and cleaved caspase-3 in control ECA-109 cells, while obviously reduced BCL-2 level and enhanced cleaved caspase-3 expression in EMS-knockdown ECA-109 cells (Figure 1I). In addition, the proliferation assay demonstrated that silencing EMS could significantly decrease the proliferation of cells treated with DDP either under normoxic or hypoxia condition (Figure 1J). Taken together, these results indicated that the lncRNA EMS can not only be upregulated by hypoxia, but also contributed to hypoxia induced DDP-resistance in EC cells.

### miR-758-3p is a downstream target of EMS in esophageal cancer cells and its downregulation contributes to cancer cell drug resistance against DDP

**Figure 2:**
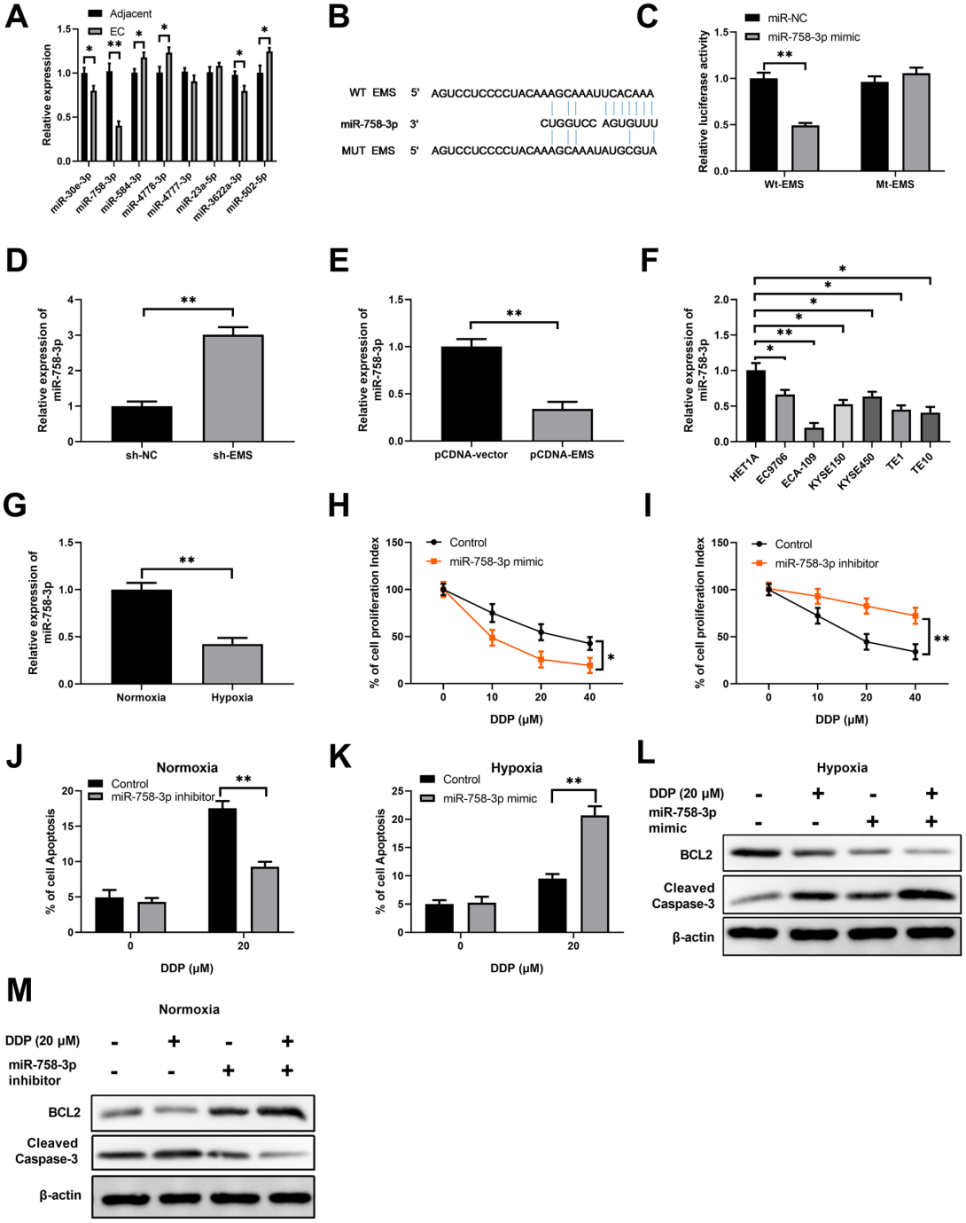
**EMS sponges miR-758-3p in EC cells, and suppression of miR-758-3p confers the resistance of EC cells to DPP.** (**A**) The expression levels of 8 possible miRNA targets in EC tumor tissues and adjacent normal tissues were determined by RT-qPCR. (**B**) Sequence comparison of miR-758-3p and wild type or mutated EMS. (**C**) Dual luciferase assay validated the interaction between EMS and miR-758-3p in ECA-109 cells. (**D**, **E**) qPCR determined the miR-758-3p expression levels in ECA-109 cells after EMS knock-down (**D**) or over-expression (**E**). (**F**) qPCR determined the miR-758-3p expression levels in indicated esophageal cancer cell lines and normal cell line HET1A. (**G**) qPCR results demonstrate that miR-758-3p expression level was significantly decreased in hypoxic ECA-109 cells, in comparison to that of normoxic ECA-109 cells. (**H**, **I**) The proliferation of ECA-109 cells after control transfection or transfection of miR-758-3p mimic (**H**) or miR-758-3p inhibitor (**I**) and in response to DDP at the indicated concentrations was determined by CCK-8 assays. (**J**–**M**) Overexpression of miR-758-3p mediated by mimic transfection in ECA-109 cells significantly increased hypoxic EC cell apoptosis rates in response to DDP, as revealed by Annexin -V flow staining (**K**); and decreased BCL2 level and increased cleaved caspase-3 level, as revealed by western blot analysis (**L**). Downregulation of miR-338-5p mediated by inhibitor transfection in ECA-109 cells significantly decreased normoxic EC cell apoptosis under DDP treatment, as revealed by Annexin V flow staining (**J**), and increased BCL2 level and decreased cleaved caspase-3 level, as revealed by western blot analysis (**M**). Representative band images from 5 independent experiments with similar results are shown, and n=5 for each group.

Next, we predicted the potential miRNA targets of EMS using the online tools, and evaluated the expression levels of 8 possible miRNA targets in EC tumor tissues and adjacent normal tissues. We found that miR-758-3p was the miRNA target with the lowest expression levels in EC tumor tissues among the 8 candidates (Figure 2A), which indicated that miR-758-3p was more likely to be regulated by EMS. Moreover, bioinformatics analysis showed that EMS could interact with miR-758-3p (Figure 2B). Therefore, we focused on miR-758-3p to study its role in hypoxia induced DDP-resistance. Indeed, the dual luciferase assays showed that the luciferase activities were markedly downregulated when wild type EMS (WT-EMS) and miR-758-3p mimics were co-transfected, but remained almost unchanged after when mutated EMS (MUT-EMS) and miR-758-3p mimics were co-transfected (Figure 2C), which suggested that EMS can regulate miR-758-3p expression through directly binding. Moreover, the lentivirus mediated knockdown of EMS resulted in remarkable increase in miR-758-3p expression in ECA-109 cells (Figure 2D), while overexpression of EMS led to substantial downregulation of miR-758-3p (Figure 2E). In addition, in contrast to bearing higher levels of EMS, all the tested human EC cell lines had significantly lower expressions of miR-758-3p than the normal esophageal epithelial HET1A cells. Consistently, ECA-109 cells with the highest abundance of EMS expression displayed the lowest level of miR-758-3p (Figure 2F).

Furthermore, hypoxia caused significantly downregulated miR-758-3p expression in ECA-109 cells in comparison to normoxia (Figure 2G), which suggests that miR-758-3p was able to against hypoxia associated DDP-resistance. This notion was further supported by the fact that while the miR-758-3p mimic inhibited the proliferation of ECA-109 cells upon treatments with DDP at various concentrations (Figure 2H), miR-758-3p inhibition with the inhibitor enhanced the proliferation of DDP-challenged ECA-109 cells under the hypoxic condition (Figure 2I). The treatment with miR-758-3p inhibitor in ECA-109 cells challenged with 20 μM DDP under the normoxic condition significantly reduced apoptosis rates (Figure 2J and Supplementary Figure 2A), whereas miR-758-3p mimic remarkably enhanced the apoptosis of DPP-treated ECA-109 cells under the hypoxic condition (Figure 2K and Supplementary Figure 2B). In concordance with these results, functional activation of miR-758-3p with mimics further reduced DDP mediated BCL-2 expression and increased DDP mediated cleaved caspase-3 level in hypoxia-insulted ECA-109 cells (Figure 2L), while functional inactivation of miR-758-3p with inhibitors caused the opposite modulation on BCL-2 and cleaved caspase-3 expressions in ECA-109 cells under the normoxic condition (Figure 2M). Collectively, these data suggested that downregulation of miR-758-3p contributed to drug resistance against DDP in EC cells.

### WTAP targeted by miR-758-3p in esophageal cancer cells is required for hypoxia-mediated resistance to DDP

**Figure 3:**
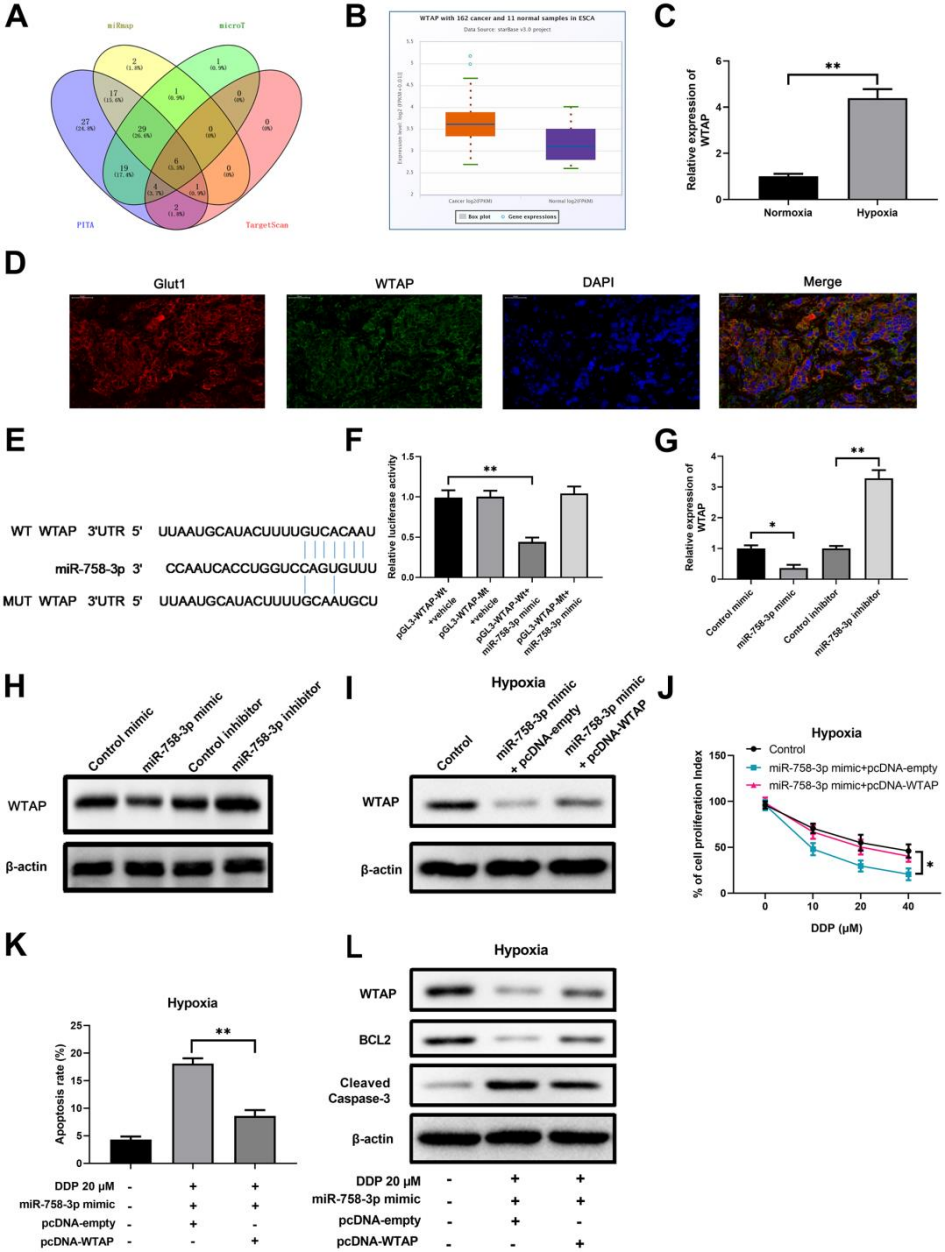
**WTAP is a downstream target of miR-758-3p in EC cells, and is required for hypoxia-mediated resistance to DDP.** (**A**) Prediction with multi-database identified 14 possible target mRNAs of miR-758-3p. (**B**) Higher expression of WTAP in esophageal cancer cells was validated using the starBase. n=162 for the cancer group; n=11 for the control group. (**C**) The WTAP mRNA expression levels in ECA-109 cells under the hypoxic and normoxic conditions were determined by RT-qPCR. (**D**) Expression of WTAP and Glut1 in EC tissue determined using immunofluorescence. (**E**) Sequence comparison of miR-785-3p and wild type or mutated 3’UTR of WTAP. (**F**) Dual luciferase assay validated the interaction between miR-758-3p and WTAP in ECA-109 cells. (**G**, **H**) qPCR assays and western blot assays were used to determine the mRNA levels (**G**) and protein levels (**H**) of WTAP in ECA-109 cells after transfection of miR-758-3p mimic/inhibitor or the corresponding controls. (**I**) Western blot results show the protein levels of WTAP in hypoxic ECA-109 cells with the indicated treatments. (**J**) Ectopic WTAP expression reversed the growth inhibitory effect of DDP in combination with miR-758-3p mimic under hypoxic condition. The proliferation of hypoxic ECA-109 cells after the indicated treatment was evaluated by CCK-8 assays. (**K**, **L**) Ectopic WTAP expression reversed the apoptosis-promoting effect of DDP in combination with miR-758-3p mimic under hypoxic condition. The apoptosis rates (**K**) and western blot results on WTAP/BCL2/cleaved-caspase-3 levels (**L**) are shown. Representative band images from 5 independent experiments with similar results are shown in J. n=5 for each group.

To identify the target gene of miR-758-3p, we employed multiple algorithms including miRmap (https://mirmap.ezlab.org/), microT (https://bio.tools/DIANA-microT), PITA and TargetScan (http://www.targetscan.org), and predicted the mRNA targets based on the presence of miR-758-3p recognition sites on the 3' UTRs. A total of 14 candidate mRNAs were predicted to be targeted by miR-758-3p (Figure 3A). We found that WTAP is one of the common candidates that were revealed by multiple algorithms. In addition, higher expression of WTAP in esophageal cancer cells was validated using the GEO database (Figure 3B). More importantly, the mRNA level of WTAP in ECA-109 cells was significantly higher in the hypoxic condition than that in the normoxic condition (Figure 3C). Additionally, a histological double staining results showed that WTAP expression was positively correlated with the hypoxia marker Glut1 expression in clinical EC samples, suggested that WTAP might play an important role in the development of EC under hypoxia condition (Figure 3D). These results support that WTAP is a potential target of miR-758-3p, which shares seven nucleotide binding sequences with the 3' UTR of WTAP (Figure 3E). Subsequent luciferase reporter assay showed that only the co-transfection of miR-758-3p mimic and the plasmid expressing wild type 3' UTR of WTAP, but not the co-transfection of the control mimics or the plasmid expressing mutated 3' UTR of WTAP, resulted in significant decrease of luciferase activity, which confirmed the binding of miR-758-3p to WTAP 3' UTR (Figure 3F). Moreover, the results of gene expression assays including RT-qPCR and Western blotting experiments showed that WTAP mRNA and protein expression levels decreased in miR-758-3p mimic-transfected hypoxic ECA-109 cells (Figure 3G) and increased in miR-758-3p inhibitor-transfected normoxic ECA-109 cells (Figure 3H).

To validate the axis of miR-758-3p/WTAP axis and the upstream and downstream regulatory relationship in human ECs, we set out to determine whether the ectopic expression of WTAP can affect the viability and apoptosis of miR-758-3p activated hypoxic ECA-109 cells. We first confirmed that down-regulated expression of WTAP protein in miR-758-3p mimic transfected ECA-109 cells was reversed upon additional transfection of WTAP-expressing plasmid (Figure 3I). Under the hypoxic condition, the decreased proliferation index in miR-758-3p mimic transfected ECA-109 cells upon challenge with various concentrations of DDP was also significantly attenuated after co-transfection of WTAP-expressing plasmid (Figure 3J). In addition, miR-758-3p mimic caused cell apoptosis in response to DDP was also reduced upon additional overexpression of WTAP, as evidenced by the flow cytometry assay-determined apoptosis rate (Figure 3K and Supplementary Figure 2C) and western blot assay-determined BCL-2 and cleaved caspase-3 protein levels (Figure 3L). Thus, WTAP is a downstream target of miR-758-3p in esophageal cancer cells, and is required for hypoxia-mediated resistance to DDP.

### Hypoxia induces WTAP expression in esophageal carcinoma to confer the resistance of cancer cells to DDP

**Figure 4:**
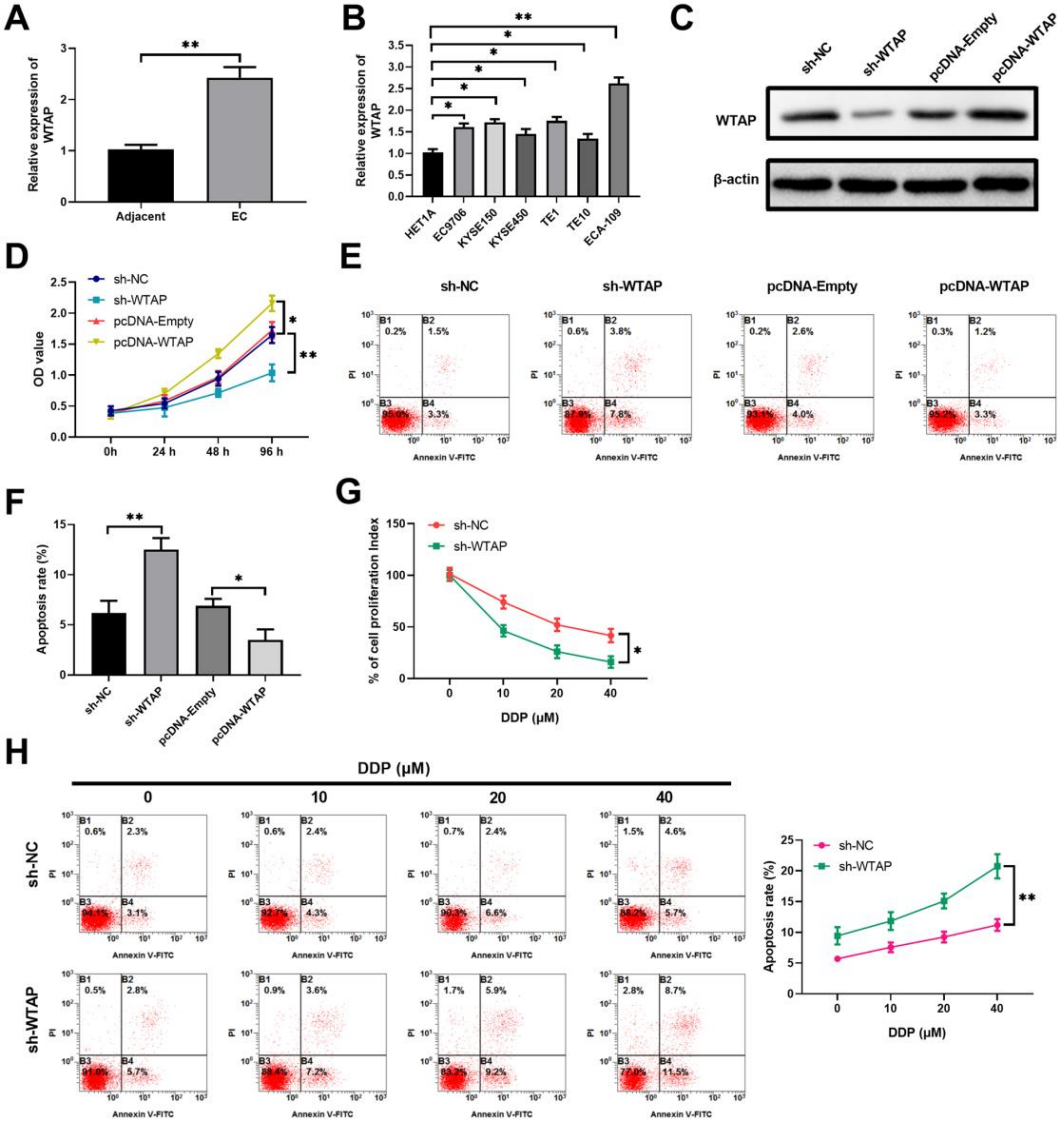
**As an oncogenic gene, WTAP is more expressed in EC cells than that in normal cells, and regulates cell proliferation and apoptosis under normoxic and hypoxic conditions.** (**A**) The expression levels of WTAP in esophageal cancer tissues and the adjacent normal tissues were determined by RT-qPCR, n=20 for each group. (**B**) Compared with normal esophageal epithelial HET1A cell, esophageal cancer cell lines (EC9706, ECA-109, KYSE150, KYSE450, TE1 and TE10) expressed more WTAP transcript, as revealed by RT-qPCR assays. n=5 for each group. (**C**) Over-expression or knockdown of WTAP in ECA-109 cells was confirmed by western blot assays at 48 hours after the indicated treatments. n=5 for each group. (**D**) Cell proliferations of indicated cells at 96 hours after treatments were measured with CCK-8 assays during a period of 4 days growth. Here, comparisons were performed between sh-NC vs. sh-WTAP and pcDNA-Empty vs. pcDNA-WTAP, respectively. n=5 for each group. (**E**, **F**) The representative flow profile of Annexin V/PI staining for indicated cells at 48 hours after treatments are shown (**E**), and summarized results on apoptosis are shown in the bar graph (**F**). n=5 for each group. (**G**, **H**) WTAP knockdown diminished the drug resistance of ECA-109 cells to DDP under hypoxic condition. The results on cell proliferation, as revealed by CCK-8 assays (**G**), and apoptosis, as revealed by annexin V-flow staining (**H**), are shown. n=3 for each group.

Since overexpression of WTAP markedly reversed the anti-tumor effects of miR-758-3p in hypoxic ECA-109 cells, WTAP is a putative oncogene that promotes esophageal cancer progression. To further explore the functions of WTAP in EC pathogenesis, we determined the expression levels of WTAP transcript in clinical samples and human EC cell lines. As expected, compared to the adjacent normal tissues, EC tumor tissues had over 2-fold expression of WTAP mRNA (Figure 4A). All the tested human EC cell lines had significantly higher levels of WTAP mRNA than the normal esophageal epithelial cell line HET1A (Figure 4B). To confirm the functional roles of WTAP in controlling EC cell proliferation and apoptosis, we manipulated the expression levels of WTAP in EC-109 cells through transducing WTAP-specific shRNA and WTAP-expressing plasmid (Figure 4C and Supplementary Figure 2D). While WTAP knockdown drastically reduced the proliferation index of normoxic ECA-109 cells, WTAP over-expression significantly enhanced cell proliferation, in comparison to the cells with control transductions (Figure 4D). Similarly, shRNA-mediated WTAP knockdown significantly increased the apoptosis of ECA-109 cells under the normoxic condition, whereas ectopic WTAP expression remarkably reduced cell apoptosis (Figure 4E, 4F). We also examined the impacts of WTAP silencing on the proliferation and apoptosis of ECA-109 cells exposed to DDP under the hypoxic condition. The hypoxic ECA-109 cells underwent a dose-dependent decrease of proliferation upon treatments with DDP, while additional WTAP knockdown further strengthened these effects (Figure 4G). Furthermore, silence of WTAP also augmented the dose-dependent killing effects of DDP against ECA-109 cells under the hypoxic condition (Figure 4H), which indicated that WTAP contributed to DDP resistance in EC cells. Taken together, as a putative oncogenic gene, WTAP is highly expressed in esophageal cancer cells and regulates cell proliferation and apoptosis under both normoxic and hypoxic conditions.

### Expression levels of the molecules in the EMS/miR-758-3p/WTAP axis in human esophageal carcinoma specimens predict patient survivals

**Figure 5:**
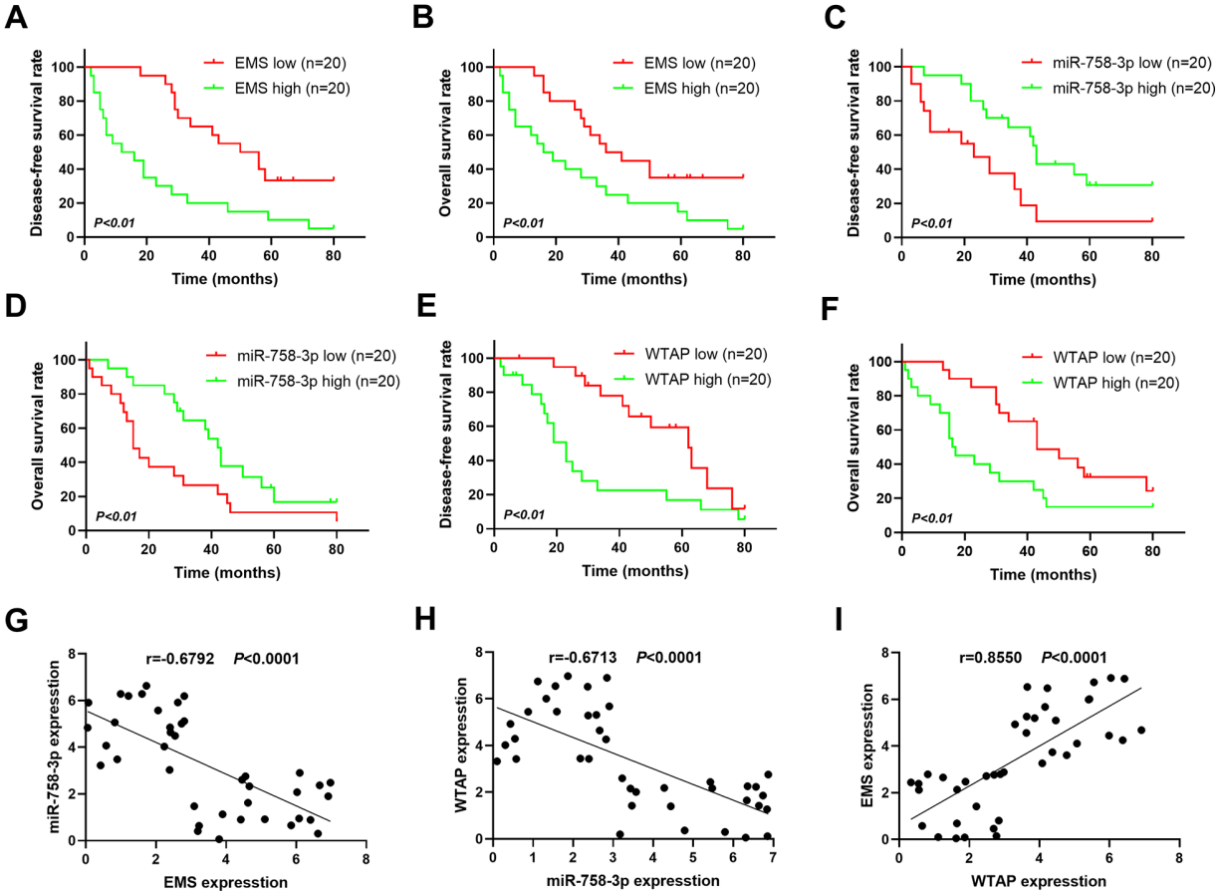
**Expressions of the EMS-miR-758-3p-WTAP axis in human EC cells predict disease progression-free survival and overall survival.** (**A**, **C**, **E**) Kaplan-Meier analysis of disease progression-free survival in patients with variable EMS (**A**), miR-758-3p (**C**), WTAP (**E**) expressions. (**B**, **D**, **F**) Kaplan-Meier analysis of overall survival in patients with variable EMS (**B**), miR-758-3p (**D**), WTAP (**F**) expressions. Based on the median value of target expression levels in tumors, these patients were categorized into expression-high and expression-low groups with 20 cases in each group. n=20 for each group. (**G**–**I**) The correlations between transcript expression levels of EMS and miR-758-3p (**G**), miR-758-3p and WTAP (**H**), EMA and WTAP (**I**) in primary human esophageal cancer tissues were analyzed.

To further decipher the clinical relevance of the EMS/miR-758-3p/WTAP axis, we examined the expression levels of EMS, miR-758-3p, and WTAP in 40 human EC specimens. Based on the median value of target expression levels in tumors, these patients were categorized into expression-high and expression-low groups with 20 cases in each group. The clinicopathological information of patients categorized to EMS-high and EMS-low groups is shown in Table 1. The patients in these two groups did not present significant differences in age and gender, but did display marked differences in terms of the Tumor, Node, Metastasis (TNM) staging and lymph node metastasis. The patients with high expression levels of EMS in tumors had significantly chances of disease progression to late TNM stages and lymph node metastasis (Table 1). In addition, Kaplan-Meier curve analysis showed that the disease-free survival (DFS; Figure 5A) and overall survival (OS; Figure 5B) of the patients with low EMS expression was obviously longer than that of patients with high EMS expression. On the contrary, the patients with lower miR-758-3p expression in their tumor tissues displayed a significantly shortened DFS (Figure 5C) and OS (Figure 5D). As expected, since WTAP is a putative oncogene in ECs, lower expression of WTAP in tumors rendered longer DFS (Figure 5E) and OS (Figure 5F) in these EC patients. Moreover, a significantly negative correlation between the expressions of EMS and miR-758-3p in tumors (Figure 5G), and the expressions of miR-758-3p and WTAP in tumors (Figure 5H), were observed, while tumor expressions of EMS and WTAP were found to be positively correlated (Figure 5I). In summary, these results demonstrated that the EMS/miR-758-3p/WTAP axis also actively existed in clinical samples, and the expression levels of these molecules can be used to predict DFS and OS in EC patients.

**Table 1:** Association between the lncRNA EMS expression and clinicopathological characteristics of patients with esophageal cancer.

**Characteristic**	**EMS expression**		**P-value**
	**Low (n=20)**	**High (n=20)**	
Age, years			
≤60	8	10	0.5250
>60	12	10	
Sex			
Male	11	12	0.7491
Female	9	8	
TNM stage			
I+II	13	3	0.012
III+IV	7	17	
Lymph node metastasis			
Negative	13	4	0.004
Positive	7	16	

### Modulation in expression levels of the molecules in the EMS/miR-758-3p/WTAP axis sensitizes cancer cells to DDP in an esophageal carcinoma xenograft model

**Figure 6:**
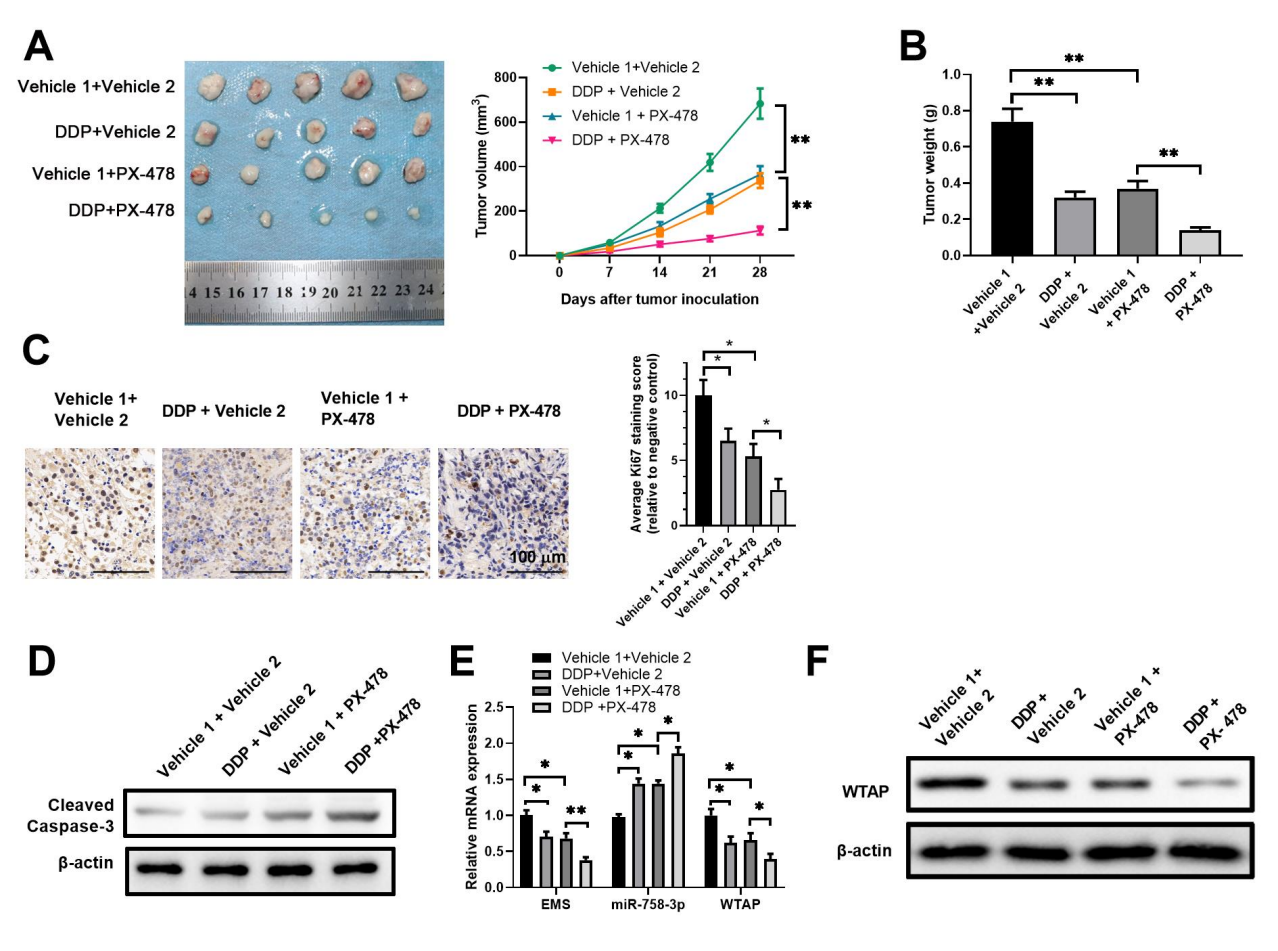
**Targeted manipulation on expression levels of the EMS/miR-758-3p/WTAP axis sensitized EC cells to DDP in an ECA-109 xenograft model.** (**A**–**F**) Nude mice were inoculated with ECA-109 cells, and were subjected to the treatments with DDP alone, the HIF-1α inhibitor PX-478 alone, or the combination of DDP and PX-478. The *in vivo* therapeutic effects, as well as the impact of these treatments on the expression levels of the molecules in the EMS-miR-758-3p-WTAP axis were evaluated. Tumor growth curves (**A**), tumor weights at the end point (**B**), cell proliferation as revealed by Ki-67 IHC staining (**C**), and apoptosis as revealed by western blot analyses of cleaved caspase-3 expression (**D**) of tumor tissues, as well as the RNA/transcript levels of EMS, miR-758-3p, and WTAP (**E**), and the protein level of WTAP (**F**) in tumors are shown. Vehicle 1, 0.9% NaCl; Vehicle 2, PBS.

To investigate the *in vivo* role of the EMS/miR-758-3p/WTAP axis in modulating chemoresistance of human ECs, we established an EC tumor xenograft animal model by subcutaneously implanting ECA-109 cells into athymic nude mice. First, we explored the impacts of DDP treatment alone, the HIF-1α inhibitor PX-478 treatment alone, and the combined treatments with DDP and PX-478, on *in vivo* tumor growth, tumor cell apoptosis, and expression levels of EMS, miR-758-3p and WTAP in tumor tissues. While either DDP alone or PX-478 alone significantly slowed down tumor growth, the combinational use of DDP and Px-478 further retarded tumor growth (Figure 6A), which resulted in further lowered tumor weight at the end point of 4 weeks after ECA-109 inoculation (Figure 6B). Using immunohistochemistry (IHC) analyses, we found a similar trend of Ki-67 expression in tumor tissues, as the combo of DDP and PX-478 further more reduced tumor Ki-67 expression than the single use of either drug (Figure 6C). In addition, compared with the control vehicle treatments, either DDP alone or PX-478 alone increased the protein level of cleaved caspase-3 in tumor tissues, which was further increased by the simultaneous treatment with DDP and PX-478 (Figure 6D). It is worth noting that the tumor tissues from the mice treated with DDP alone or PX-478 alone had significantly reduced expressions of EMS and WTAP, as well as significantly increased expression of miR-758-3p, in comparison to the tumor tissues from control vehicles treated mice, whereas combined DDP and PX-478 treatments further enhanced these alterations (Figure 6E). Consistently, the protein levels of WTAP in tumor tissues also demonstrated a similar pattern as its mRNA levels (Figure 6F). Therefore, these results demonstrated the association between the expression levels of molecules in the EMS/miR-758-3p/WTAP axis and tumor progression under the chemotherapy and hypoxia disruption conditions, and further suggested the potential of modulating the EMS/miR-758-3p/WTAP axis for sensitizing EC cells.

**Figure 7:**
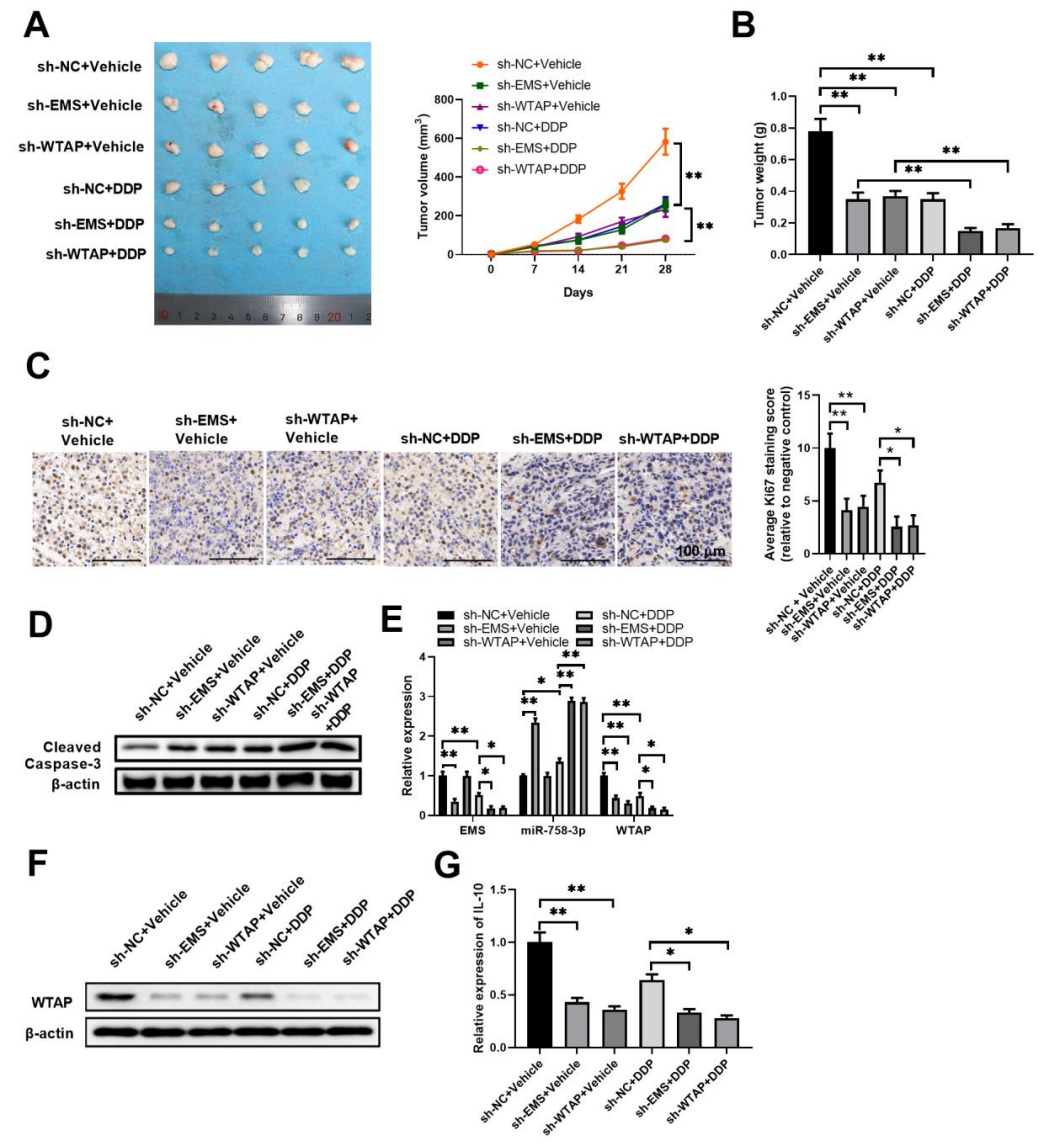
**Targeted knockdown of EMS or WTAP significantly enhanced the therapeutic effects of DDP in tumor eradication.** Nude mice were inoculated with EMS-silenced or WTAP-silenced ECA-109 cells and treated with DDP. Tumor growth curves (**A**), tumor weight (**B**), cell proliferation (**C**), and apoptosis (**D**) of tumor tissues, as well as the RNA/transcript levels of EMS, miR-758-3p, and WTAP (**E**), the protein level of WTAP (**F**), and mRNA levels of mouse IL-10 (**G**) in tumor tissues are shown. For tumor volume comparisons (**A**), sh-NC+Vehicle, sh-EMS+Vehicle, or sh-WTAP+Vehicle group was compared with their corresponding DDP treatment group, respectively. n=5 mice in each group, and representative data are from at least 5 experiments with similar results. n=5 for each group.

To test this notion, we implanted EMS-silenced or WTAP-silenced ECA-109 cells to nude mice, and examined their responses to DDP challenge *in vivo*. As shown in Figure 7A, 7B, among the groups without DDP treatments, either EMS knockdown alone or WTAP knockdown alone could significantly reduce tumor sizes and tumor weights. Among the DDP treated groups, compared with the control treatment group, although DDP treatment alone slowed down tumor growth, either EMS knockdown alone or WTAP knockdown alone further reduced tumor sizes and tumor weights. Likewise, the reduced expression of Ki-67 in tumor tissues was most evident in the groups with EMS/WTAP knockdown and DDP treatments (Figure 7C). Moreover, western blot assays also revealed the most expression of cleaved caspase-3 in tumor tissues from the groups with EMS/WTAP knockdown and DDP treatments (Figure 7D). Furthermore, subsequent RT-qPCR (Figure 7E) and western blot (Figure 7F) analyses confirmed that the differences in *in vivo* anti-tumor efficacies among the groups were tightly associated with the expression levels of the molecules in the EMS/miR-758-3p/WTAP axis. Since upregulated WTAP expressions have been revealed to be significantly associated with immunosuppression in malignancies like human hepatocarcinoma and gastric cancer [24, 25], we also examined the mRNA levels of mouse immunosuppressive cytokine IL-10 in our model. As shown in Figure 7G, the expressions of IL-10 mRNA in tumor tissues among groups displayed a similar trend as that of WTAP, which verified the correlation between WTAP expression and immunosuppression in our xenograft animal model. Taken together, we confirmed that modulation on the expression of the EMS/miR-758-3p/WTAP axis could alter the *in vivo* sensitivity of EC cells to DDP therapy.

## DISCUSSION

Esophageal carcinoma remains one of the leading causes of cancer-related death, while chemoresistance is a challenging obstacle in EC therapy, which is urgently needed to be resolved [4]. Previous studies have demonstrated that hypoxia in the tumor environment contributes significantly to the development of chemoresistance [23]. Recently, aberrant expressions of lncRNAs were revealed to be associated with various malignant behaviors of tumor cells including chemoresistance [19]. However, little is known on the regulatory mechanisms of lncRNAs in the context of hypoxia mediated chemoresistance in EC. In the present study, we found that a novel lncRNA EMS was hypoxia-induced and overexpressed in human EC tumor tissues and cell lines. Importantly, the expression of EMS was required for hypoxia-mediated drug resistance to DDP in the ECA-109 cell line. Mechanistically, we further identified a lncRNA-EMS/miR-758-3p/WTAP axis involving hypoxia induced downregulation of miR-758-3p expression and upregulation of WTAP expression that contributed substantially to DDP resistance in ECA-109 cells, in terms of controlling cell proliferation and apoptosis. In addition, higher EMS/WTAP or lower miR-758-3p expressions in tumors can predict worse survivals (both PFS and OS) of EC patients. Furthermore, treatments with DDP and the HIF-1α inhibitor PX-478 in the human EC xenograft animal model confirmed that the tumor expression levels of the molecules in the lncRNA-EMS/miR-758-3p/WTAP axis were associated with tumor rejection efficiencies. Importantly, targeted knockdown of EMS or WTAP significantly sensitized the ECA-109 tumor cells to *in vivo* DDP therapy in the xenografted mouse model, which implied the values of these molecules as potential therapeutic targets in EC treatments.

Malignant cells can develop the ability of chemotherapy resistance prior to and following exposure to anti-cancer drugs [23]. Increasing evidence has revealed that hypoxia linked HIF-1α overexpression is found to be associated with lymph node metastasis and pathologic stage, poor EC differentiation and increased depth of tumor invasion, disease-free survival rate, and initial response to concurrent chemoradiotherapy [26–29]. Consistent with previous studies, we demonstrated that hypoxia rendered drug resistance to DDP in ECA-109 cells, as evidenced by drastically high DDP IC_50_, which was largely mediated by the activation of the lncRNA-EMS/miR-758-3p/WTAP axis. Compared to the normal esophageal epithelial cell line HET1A, all the EC cell lines displayed significantly higher expressions of EMS and WTAP, as well as significantly lower expressions of miR-758-3p. Similarly, in clinical samples from EC patients, the more hypoxic tumor tissues had the same expression patterns of these molecules over the less hypoxic adjacent normal tissues. Moreover, *in vivo* PX-478 treatment also markedly reduced the expressions of EMS and WTAP, and elevated the expressions of miR-758-3p in xenografted ECA-109 cells. Taken together, these results substantiated the roles of the lncRNA-EMS/miR-758-3p/WTAP axis in conferring hypoxia-mediated drug resistance to DDP in EC cells. Furthermore, in our xenograft mouse model, combinational use of DDP and PX-478 not only more effectively eradicated tumors, but also further altered the tumor expression patterns of the participating molecules in the axis, compared to either single use of these drugs. This supports that a combination treatment with DDP and PX-478 can effectively reverse the drug resistance, and provides the rationale to combine DDP and PX-478 or other HIF-1α inhibitors for the clinical EC therapy.

LncRNAs have been identified as significant players in gene regulation with the context-dependent molecular mechanisms [19]. The novel lncRNA EMS was identified as a direct c-Myc transcriptional target that functions as an oncogenic gene by increasing E2F1 expression and promoting G1/S cell cycle progression in lung cancer cells [22]. In this study, we extended the oncogenic role of EMS in esophageal cancer cells, from a prospective of conferring hypoxia-associated chemoresistance. To our knowledge, this is the first report on revealing the oncogenic role of EMS in a tumor type other than lung cancer. Since HIF-1α and c-Myc act in concert in reprograming metabolism, protein synthesis and cell cycle progression to fine tune adaptive responses of tumors to the hypoxic environments [30], whether a similar connection of EMS and c-Myc in regulating hypoxia-associated chemoresistance also exists in EC remains to be elucidated in future. Alternatively, as DDP is also widely used in chemotherapy of lung cancer patients, whether the lncRNA-EMS/miR-758-3p/WTAP axis applies to the DDP resistance in lung cancer therapy awaits for further studies. A series of miRNAs have been identified to function in the development of drug resistance [31]. Here, we identified miR-758-3p as the sponging target of EMS, and suppression of miR-758 conferred the resistance of EC cells to DPP. miR-758-3p was reported to suppress the progression of many cancer types, including bladder cancer, papillary thyroid cancer, acute myeloid leukemia, ovarian cancer, gastric cancer, non-small cell lung cancer, and hepatocellular carcinoma [32–38]. However, the roles of miR-758-3p in the progression of esophageal cancer have not been studied. Consistent with previous reports, our results also suggest a tumor suppressor role for miR-758-3p in EC. miR-758-3p was downregulated in EC tissues compared with that in matched normal tissues, and its expression negatively correlated with the malignant phenotypes of ECA-109 cells in terms of cell proliferation and apoptosis. It is worth noting that miR-758-3p was selected for further investigation due to its lowest expression level in EC tissues among the 8 candidate miRNAs, whereas other two miRNAs including miR-30e-3p and miR-3622a-3p also demonstrated downregulated expressions. Given the fact that one lncRNA can targets many miRNAs [39], and considering the chemoresistance properties and tumor suppressive effects of miR-30e and miR-3622a [40, 41], whether and how these two miRNAs interact with EMS and contribute to DDP resistance in EC cells also deserves further elucidation.

The multi-database prediction and the subsequent luciferase-based reporter assays identified that WTAP is a target of miR-758-3p. As a nuclear protein that specifically interacts with WT1, WTAP is ubiquitously expressed in different tissues and functions in biological processes [42]. We found that WTAP expression was positively correlated with the malignant phenotypes of ECA-109 cells including active proliferation and reduced apoptosis under the hypoxic condition. Importantly, WTAP was found to be required for hypoxia-mediated resistance to DDP in EC cells. Apparently, WTAP functions as a tumor-promoting factor in the pathogenesis of human esophageal cancer. Indeed, WTAP expression has been connected to couples of malignancies, such as ovarian cancer, hepatocellular carcinoma, pancreatic cancer, bladder cancer, diffuse large B-cell lymphoma, renal cell carcinoma, and pancreatic ductal adenocarcinoma [43–49]. In accordance with our finding that higher levels of tumor WTAP expression predicted worse survival rates of EC patients, WTAP is also a prognostic marker for high-grade serous ovarian cancer [43], bladder cancer [50], glioma [51], and pancreatic ductal adenocarcinoma [52]. Notably, a recent report from Li et al revealed that WTAP could promote migration/invasion and suppress chemosensitivity to gemcitabine in pancreatic cancer [45]. This study implies that WTAP might contribute to a broad spectrum of chemotherapy drugs in multiple cancer types. In supporting of the contribution of WATP in conferring chemoresistance, our ECA-109 xenograft mice with reduced tumor burden all displayed markedly decreased expressions of WTAP mRNAs and proteins, and WTAP knockout in tumors significantly attenuated the resistance to DDP treatment. It is worth noting that WTAP knockout did not result in complete eradication of DDP-challenged tumors. This might be due to the inadequate knockdown of WTAP, or due to that other downstream molecules rather than WTAP also account for DDP resistance. For instance, whether other candidate targets of miR-758-3p, such as NOTCH2 [32], TAB1 [33], CBX5 [36], and MDM2 [38], were also involved in DDP resistance in EC cells remains to be further investigated. Nevertheless, the therapeutic strategies targeting WTAP are very promising for more effectively treating patients with chemo-resistant EC.

The current study has couples of strengths and limitations. As we are aware, this is the first-time report on revealing the important role of EMS in hypoxia mediated chemo-resistance of human esophageal cancer. This is also the first report on linking the function of miR-758-3p, as well as that of WTAP, to the pathogenesis of EC. It is novel that WTAP expression in EC was identified to be upregulated under the hypoxic condition, which contributed to the chemoresistance to cisplatin in EC cells. The current work also suggests a feasible framework to explore chemo-resistance-related potential small RNA and mRNA molecules to enhance the strategies for better diagnosis and therapy of patients with various cancer types. One limitation of the current work is the sample size of clinical tissues and data sources in evaluation of the expression levels of the participating members of the lncRNA-EMS/miR-758-3p/WTAP axis in tumor and non-tumor tissues, as well as calculating the survival rates of EC patients. Moreover, more work on substantiating the therapeutic effects of *in vivo* silence of EMS or WTAP, as well as the *in vivo* delivery of miR-758-3p mimics, in EC xenografted animals, might be needed to consolidate the potential of the lncRNA-EMS/miR-758-3p/WTAP axis-targeted therapy in EC patients.

In conclusion, the present study unveiled a lncRNA-EMS/miR-758-3p/WTAP axis underlying the hypoxia-mediated drug resistance to DDP in EC cells, and our results suggest that each molecule of this axis could be considered as a novel potential target to attenuate chemoresistance in EC patients. Our data not only reveal a role of the lncRNA-EMS/miR-758-3p/WTAP axis in cancer chemo-resistance, but also provide a novel insight in the diagnosis, therapy, and prognosis of human esophageal cancer.

## MATERIALS AND METHODS

### Cell lines and clinical tissue samples

Human normal esophagus epithelial cell line HET1A and human esophageal cancer cell lines ECA-109, EC9706, KYSE150, KYSE450, TE1, and TE10 were bought from Cell Bank of Chinese Academy of Sciences (Shanghai, China). Cells were cultured in DMEM supplemented with 10% fetal bovine serum (FBS), 100 units/ml of penicillin and 100 μg/ml of streptomycin (all from Thermo Fisher Scientific, Waltham, MA, USA). Cells were maintained in a humidified incubator with 5% CO_2_ at 37° C. For hypoxia *in vitro* model establishment, cells were maintained in conventional DMEM and cultured in a humidity cell incubator at 37° C with atmosphere composition of 2% O_2_, 93% N_2_, and 5% CO_2_ for 24 h.

Patients with esophagus carcinoma were recruited between 2015 and 2019 in Gansu Provincial People's Hospital (Lanzhou City, China). Tumor samples and adjacent normal esophagus epithelial tissue samples (n = 40 pairs) were gathered from the patients who underwent surgical resection in Gansu Provincial People's Hospital. After immediately freezing in liquid nitrogen, resected tissues were stored at −80° C. Written informed consent was obtained from all the patients or their guardians. Ethics clearance was obtained from Institutional Center Ethics Review Committee in Gansu Provincial People's Hospital.

### Cell treatments, gene knockdown and over-expression

The chemotherapy drug cisplatin (DDP) was procured from Sigma-Aldrich (St. Louis, MO, USA), and the HIF-1α inhibitor PX-478 was bought from Selleck (Houston, TX, USA). For gene over-expression, the DNA coding sequences for EMS and WTAP were cloned into the mammalian expression vectors pcDNA3.1 (Thermo Fisher Scientific). For gene knockdown, DNA sequences corresponding to the short hairpin RNA (shRNA) specific for EMA and WTAP as well as the non-specific shRNA were cloned into the lentivirial vector (Ribobio, China). The mimic and inhibitor of miR-785-3p, and their corresponding negative controls (miR-NC and inhibitor-NC) were synthesized by Shanghai GenePharma Co., Ltd. (Shanghai, China). ECA-109 cells were infected by lentivirus or transfected with the indicated plasmids, miRNA mimics or inhibitors with Lipofectamine 3000 (Thermo Fisher Scientific) following the manufacturer's instructions. At 72 hours after transfection, cells were harvested for experiments. The sequences of all mimics, inhibitors and their negative controls are listed in the Supplementary Table 1.

### Cell proliferation and apoptosis assays

The proliferation of ECA-109 cells at 48 hours after indicated virus infection and/or miRNA mimics or inhibitors transfection was determined with the Cell Counting Kit-8 (CCK-8) assay kit (Dojindo; Kumamoto, Japan) per the manufacturer’s specifications. The absorbance of samples at 450 nm was acquired via a microplate reader (Bio-Rad Laboratories; Hercules, CA, USA), and cell proliferation index was calculated as the relative value of the absorbance of the sample to a non-treatment control. The drug half maximal inhibitory concentration (IC_50_) was calculated using the Probit regression analysis provided by the IBM® SPSS Software (version 19.0).

Cell apoptosis was measured using the apoptosis kit purchased from BD Biosciences (San Jose, CA, USA) per the manufacturer's instructions. Flow cytometry data acquired by FACSCalibur (BD Biosciences) were analyzed using the expo32 software (Beckman Coulter, Brea, CA, USA). Annexin V-FITC (fluorescein isothiocyanate) positive cells, including PI (propidium iodide) negative (early apoptosis) and positive (late apoptosis) cells, were considered as apoptotic cells.

### Luciferase reporter assay

The Dual-Luciferase Reporter Assay System (Promega, Madison, WI, USA) was used for luciferase reporter assay. Briefly, the tested candidate molecules, including wild type and mutated EMS coding sequence or the predicted wild type and mutated binding sites within the 3' UTR (un-translated region), were cloned into the luciferase-expressing vector pGL3. ECA-109 cells were then transfected with the indicated vector, miRNA inhibitor, mimics, or controls. Cells were harvested at 48 hours after transfection, and the luciferase reporter assay was carried out per the manufacturer's instructions. The luciferase activity from cells with co-transfection of miRNA control inhibitor or mimics was used for normalizations.

### Real-time RT-PCR

Total RNA and miRNA samples were obtained using the TRIzol reagent (Thermo Fisher Scientific) and the miRNeasy mini kit (Qiagen, Hilden, Germany), respectively. Reverse transcription was performed with the PrimeScript® RT Master Mix Perfect Real Time Reagent Kit (Takara Bio Inc., Shiga Prefecture, Japan) and miScript II RT kit (Qiagen) for total RNA and miRNA, respectively. Real-time quantitative PCR (RT-qPCR) was conducted using the SYBR Green PCR kit (Toyobo, Osaka, Japan) with a standard AB7500 RT-PCR instrument. RT-qPCR data were analyzed with the 2^-ΔΔCT^ method. The PCR primers used in this study are presented in Supplementary Table 2.

### Western blot assay

Cells and tissues were lysed with the radioimmunoprecipitation assay lysis buffer (RIPA; Sigma-Aldrich), and protein samples were obtained from the supernatant after centrifugation at 13000 g for 20 minutes at 4° C. Samples were separated by 10% or 15% SDS-PAGE. Proteins in samples were transferred to polyvinylidene difluoride (PVDF) membranes, and the membranes were incubated with indicated primary antibodies overnight. Anti-WTAP antibody (1:1,000 dilution; cat. no. 60188-1-Ig; Proteintech Group, Inc), anti-Bcl2 antibody (1:1,000 dilution; cat. no. 12789-1-AP; Proteintech Group, Inc), anti-cleaved caspase-3 antibody (1:1,000 dilution; cat. no. 19677-1-AP; Proteintech Group, Inc) and the antibody for the loading control β-actin (1:10,000 dilution; cat no. 60008-1-Ig; Proteintech Group, Inc) were used. After binding with the secondary horseradish peroxidase (HRP)-conjugated anti-rabbit or mouse IgG (1:10,000; cat. no SA00001-2 or cat. no SA00001-1, Proteintech Group, Inc), the membranes were processed with the Immobilon Western Chemiluminescent HRP Substrate (EMD Millipore, Burlington, MA, USA). Protein expressions were quantified by densitometry with the ImageJ software (National Institutes of Health, Bethesda, MD, USA).

### Immunohistochemistry staining

Immunohistochemistry staining of tumor tissues was performed using the routine procedures. The rabbit-anti Ki-67 antibody (1: 400 dilution; Thermo Fisher Scientific) and HRP-conjugated secondary goat-anti-rabbit monoclonal antibody (1: 200 dilution; Thermo Fisher Scientific) were used. Before being mounted, the slices were stained with 0.5% diaminobenzidine and counterstained with Mayer's hematoxylin.

### Double staining immunofluorescent (IF)

For IF analysis, frozen tumor tissue derived from EC patients were cut into 7 μm-thick slices with cryostat. Then, the slices were blocked with 5% goat serum in PBS for 30 min and incubated with rabbit anti-WTAP antibody (1:200 dilution; Thermo Fisher Scientific) followed by goat anti-rabbit antibody conjugated with Alexa Fluro 488 (1:500 dilution; Thermo Fisher Scientific) under a dark condition. After blockaded with 5% goat serum for 30 min, the slices were then incubated with mouse anti-Glut1 antibody (1:200 dilution; Thermo Fisher Scientific) and followed by the donkey anti-mouse antibody conjugated with Alexa Fluro 546 (1:500 dilution; Thermo Fisher Scientific) in a dark room. Subsequently, the slides were mounted with DAPI-containing mounting medium (Vector laboratories, Burlingame, CA, USA). A Leica fluorescence microscope (Leica, Germany) was used to take images.

### Xenograft tumor model

Nude mice (all female, 5-7 weeks old) were bought from the SLAC Laboratory Animal Ltd., Co. (Shanghai, China). Before starting experiments, mice were acclimated in a specific-pathogen-free (SPF) room at the Experimental Animal Center of Gansu Provincial People's Hospital for one week. Wild type ECA-109 cells or ECA-109 cells after infection of control-shRNA-expressing, or EMS-shRNA-expressing or WTAP-shRNA-expressing lentivirus were injected (1×10^6^ cells/mouse) into the right flank of nude mice. At 2 weeks after tumor inoculation, mice were treated with DDP (10 mg/kg body weight, dissolved in 0.9% NaCl), and/or PX-478 (20mg/kg body weight, dissolved in PBS) by intraperitoneal (i. p.) injection every other day for 4 weeks. The long diameter (*a*) and short diameter (*b*) of tumors were measured every 4 days, and the tumor volume was calculated with the following formula: v=ab^2^/2. At 4 weeks after treatments, mice were sacrificed. All the animal experimental procedures were approved by the Institutional Animal Care and Use Committee (IACUC) of Gansu Provincial People's Hospital.

### Bioinformatics analyses

Prediction of the candidate genes targeted by miR-758-3p and the binding sites of miR-758-3p was conducted using the online tools TargetScan (http://www.targetscan.org/), PITA, microT_CDS (https://tools4mirs.org/software/target_prediction/diana-microt-cds/) and miRmap (https://mirmap.ezlab.org/). The published microarray datasets representing 162 primary esophageal cancer tissues were downloaded from starBase (https://web.archive.org/web/20110222111721/http://starbase.sysu.edu.cn/). The values of Fragments Per Kilobase of transcript per Million mapped reads (FPKM) were used to quantify the expression levels of WTAP transcript.

### Statistics

The SPSS v.19.0 statistical software was used for statistical analyses. Data are shown as mean ± standard deviation (SD), and were compared using unpaired, two-tailed Student’s t-tests. Kaplan–Meier survival curves with log-rank test was used for survival analyses. The associations between the gene expression levels of two genes were determined using Pearson’s R value, the chi-square test, and Fisher’s exact test. A *P* value < 0.05 indicates statistically significant.

### Availability of data and materials

The datasets used and/or analyzed during the current study available from the corresponding author on reasonable request.

### Ethics approval and consent to participate

Written informed consent was obtained from all patients. The present study was approved by the Ethics Committee of Gansu Provincial People's Hospital. The animal experiment was approved by the Institutional Animal Care and Use Committee of Gansu Provincial People's Hospital.

## Supplementary Material

Supplementary Figures

Supplementary Tables
