# Supplementary material for: Hypoxia induces chemoresistance of esophageal cancer cells to cisplatin through regulating the lncRNA-EMS/miR-758-3p/WTAP axis
Source: Aging (Albany NY). 2021 Jun 3;13(13):17155–76. doi: 10.18632/aging.203062 (PMC8312407; doi:10.18632/aging.203062)
Supplement: Supplementary Figures [file aging-13-203062-s001.pdf]

SUPPLEMENTARY FIGURES

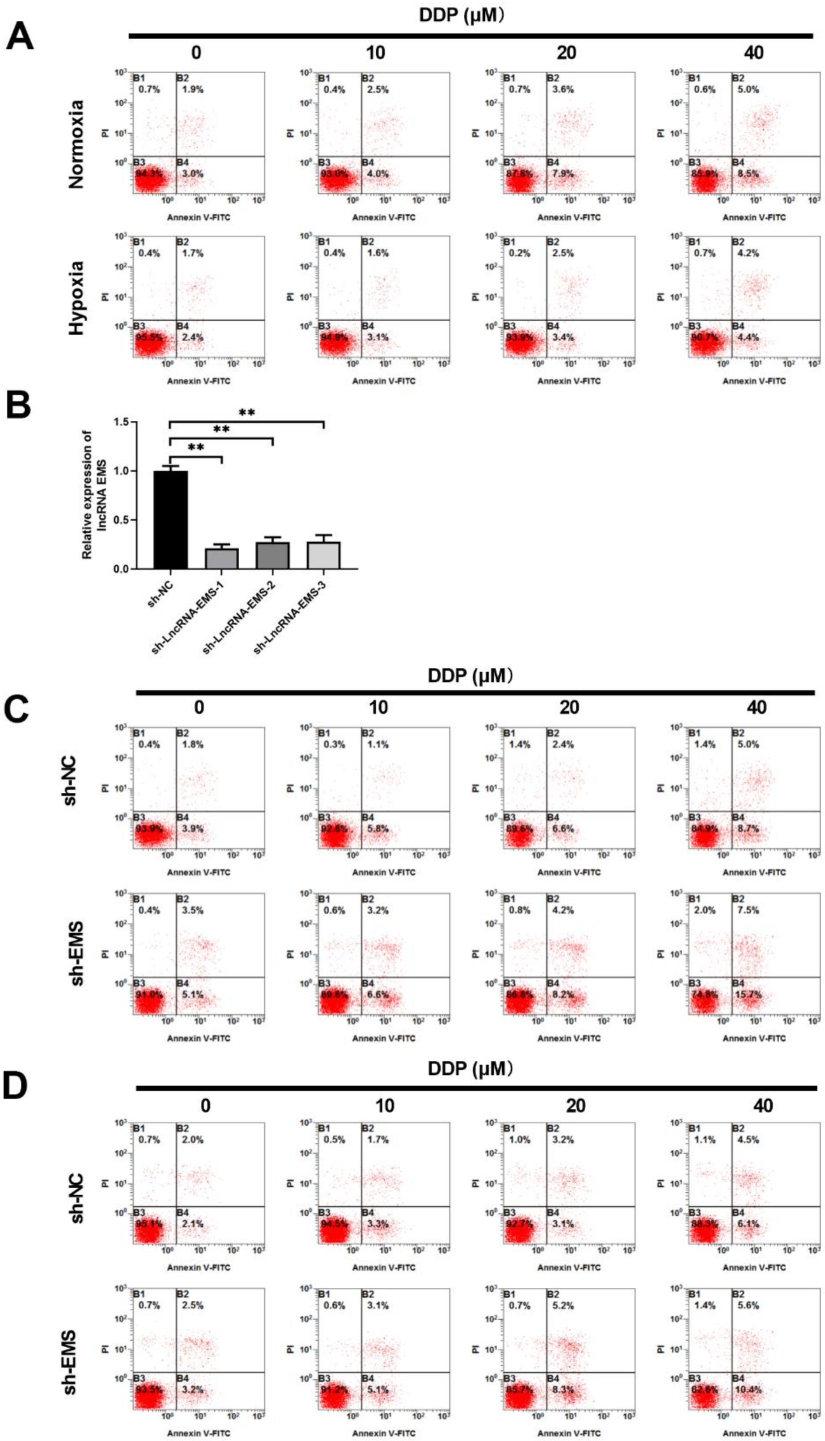

Supplementary Figure 1. Silencing lncRNA EMS increases the apoptosis of esophageal carcinoma ECA-109 cells induced by DDP both under normoxia and hypoxia. (**A**) Apoptosis of ECA-109 cells at normoxia and hypoxia conditions in the presence of DDP at the indicated doses for 24 h determined by flow cytometry. (**B**) Confirmation of lncRNA EMS knockdown in ECA-109 cells with shRNA method determined by RT-qPCR. (**C**) Apoptosis of ECA-109 cells in the presence of DDP at the indicated doses for 24 h under normoxia determined by flow cytometry. (**D**) Apoptosis of ECA-109 cells in the presence of DDP at the indicated doses for 24 h under hypoxia determined by flow cytometry.

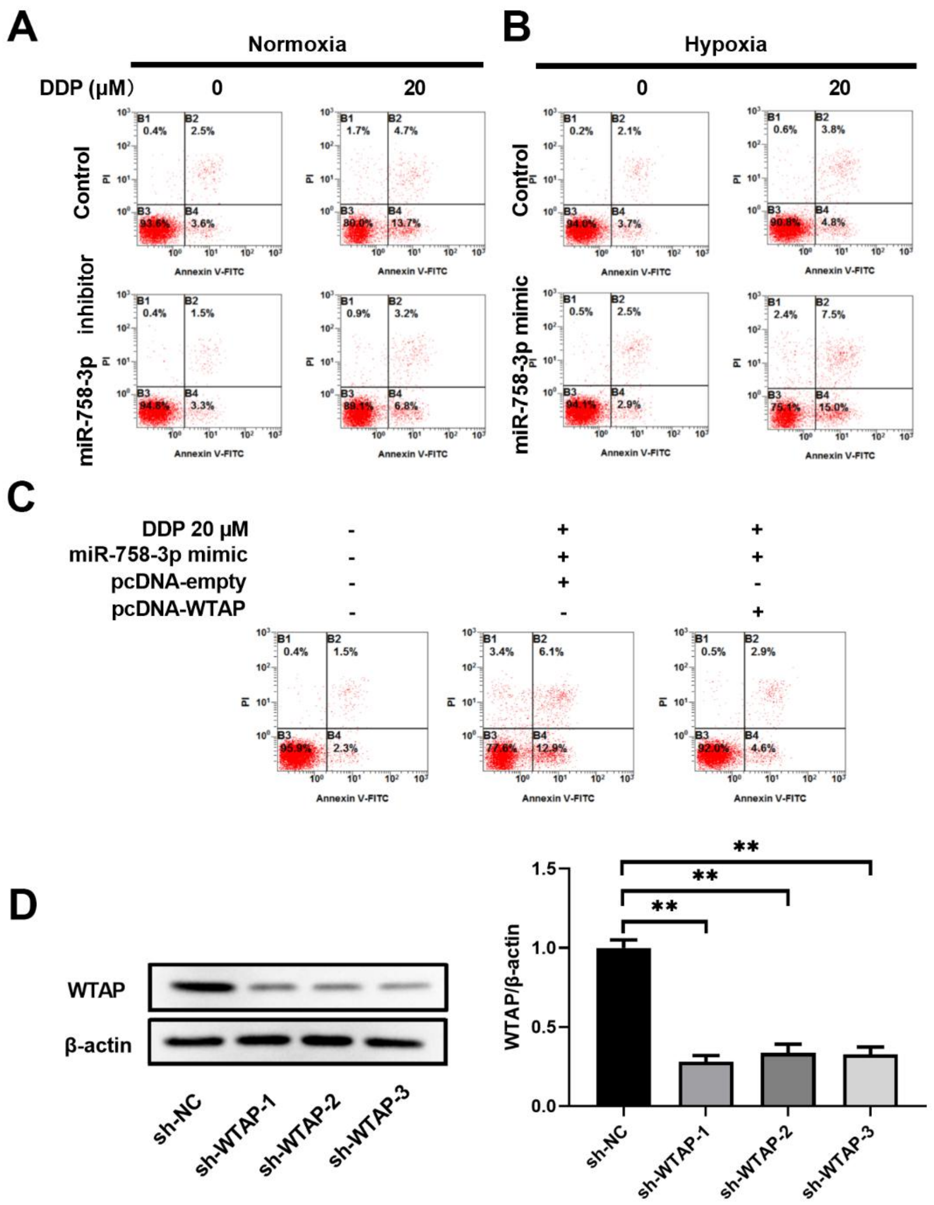

Supplementary Figure 2. WTAP alleviates the effect of miR-758-3p on the apoptosis of esophageal carcinoma ECA109 cells induced by DDP. (**A**) Apoptosis of ECA-109 cells after transfected with miR-758-3p inhibitor and treated with 20 μM DDP under normoxia; (**B**) Apoptosis of ECA-109 cells after transfected with miR-758-3p mimic and treated with 20 μM DDP under hypoxia; (**C**) Apoptosis of ECA-109 cells after transfected with miR-758-3p mimic and treated with 20 μM DDP under hypoxia. (**D**) Confirmation of WTAP knockdown in ECA-109 cells using western blot.
