# Supplementary material for: Hypoxia induces chemoresistance of esophageal cancer cells to cisplatin through regulating the lncRNA-EMS/miR-758-3p/WTAP axis
Source: Aging (Albany NY). 2021 Jun 3;13(13):17155–76. doi: 10.18632/aging.203062 (PMC8312407; doi:10.18632/aging.203062)
Supplement: Supplementary Tables [file aging-13-203062-s002.pdf]

SUPPLEMENTARY TABLES

Supplementary Table 1. The sequences of shRNA, miRNA mimics, inhibitors and negative controls used in this study.

Name	Sequences (5' - 3')
sh-NC	5′-TTCTCCGAACGTGTCACGT-3′
sh-EMS-1	5’-UGAUAGACUAGAUCAAGCA-3’
sh-EMS-2	5’-GCAGAUCCGCUAAGAGAAC-3’
sh-EMS-3	5’-UGUCAUAUAGAGAAUUGUG-3’
sh-WTAP-1	5’-GCAAGAGTGTACCACTTAAATGTTAATATTCATAGCATTTGAGTGGTGCACTCTTGCTTTTTG-3’
sh-WTAP-2	5’-CCGGATGGCAAGAGATGAGTTAATTCTCGAGAATTAACTCATCTCTTGCCATTTTTTG-3’
sh-WTAP-3	5’-CCGATTGAGTGAAACAGATTTGTTAATATTCATAGCAAGTCTGTTTCACTCAGTCGGTTTTTG-3’
miR-758-3p mimic	5’-UUUGUGACCUGGUCCACUAACC-3’
mimic NC	5′-ACAUCUGCGUAAGAUUCGAGUCUA-3′
miR-758-3p inhibitor	5′-GGUUAGUGGACCAGGUCACAAA-3′
inhibitor NC	5’-GCGUAACUAAUACAUCGGAUUCGU-3’

Supplementary Table 2. The sequences of primers for qRT-PCR analyses used in this study.

Name	Sequences (5' - 3')
U6	F: 5′-CTCGCTTCGGCAGCACA-3′<br>R: 5′-AACGCTTCACGAATTTGCGT-3′
EMS	F: CCTGCGCGGAGAAATTGGAT<br>R: GAATGGCCAGAGTACAACCCA
miR-758-3p	F: 5′-ACACTCCAGCTGGGTTTGTGACCTGGTCCA-3′<br>R: 5′-CTCAACTGGTGTCGTGGAGTCGGCAATTCAGTTGAGGGTTAGTG-3′;
miR-30e-3p	F: 5′- GGGCTTTCAGTCGGATGTT-3′<br>R: 5′-GTGCGTGTCGTGGAGTCG-3′
miR-584-3p	F: 5′-CGGCGGTCAGTTCCAGGCCAAC-3′<br>R: 5′-CTGGTGTCGTGGAGTCGGCAATTC-3′
miR-4778-3p	F: 5’-TCTTCTTCCTTTGCAGAGTTGA-3’<br>R: 5′-CTGGTGTCGTGGAGTCGGCAATTC-3′
miR-4777-3p	F: 5’-ATACCTCATCTAGAATGCTGTA-3’<br>R: 5′-CTGGTGTCGTGGAGTCGGCAATTC-3′
miR-23a-5p	F: 5’-ACACTCCAGCTGGGGGGGTTCCTGGGGA-3’<br>R: 5’-CTCAACTGGTGTCGTGGAGTCGGCAATTCAGTTGAGAAATCCCA-3’
miR-3622a-3p	F: 5’TCACCTGACCTCCCATGCCTGT-3’<br>R: 5’-GCGAGCACAGAATTAATACGAC-3’
miR-502-5p	F: 5′-ATCCTTGCTATCTGGGTGCTA-3′<br>R: 5’-CTGGTGTCGTGGAGTCGGCAATTC-3’
HPRT	F: 5‘-CATTTGTCGATGCTCAATCC-3’<br>R: 5‘-TCAGTTGAAGTCATTGATGATGG-3’
WTAP	F: 5’-GCAAGATGACCAACGAAGAAC-3’,<br>R: 5’-CCAGTCACATCGTTTGAATTAAG-3’
Mouse IL-10	F: 5’-CGGGAAGACAATAACTGCACCC-3’<br>R: 5’-CGGTTAGCAGTATGTTGTCCAGC-3’
Mouse β-actin	F: 5’-AACCGTGAAAAGATGACCCAGAT-3’<br>R: 5’-CACAGCCTGGATGGCTACGTA-3’
